# Neglected Venomous Animals and Toxins: Underrated Biotechnological Tools in Drug Development

**DOI:** 10.3390/toxins13120851

**Published:** 2021-11-29

**Authors:** Guilherme Rabelo Coelho, Daiane Laise da Silva, Emidio Beraldo-Neto, Hugo Vigerelli, Laudiceia Alves de Oliveira, Juliana Mozer Sciani, Daniel Carvalho Pimenta

**Affiliations:** 1 Laboratório de Bioquímica, Instituto Butantan, São Paulo 05503-900, Brazil; guilherme.coelho@butantan.gov.br (G.R.C.); daiane.silva@esib.butantan.gov.br (D.L.d.S.); emidio.beraldo@butantan.gov.br (E.B.-N.); 2 Laboratório de Genética, Instituto Butantan, São Paulo 05503-900, Brazil; hugo.barros@esib.butantan.gov.br; 3 Laboratório de Moléstias Infecciosas—Faculdade de Medicina de Botucatu, São Paulo State University (UNESP), São Paulo 01049-010, Brazil; laudiceia.oliveira@unesp.br; 4 Laboratório Multidisciplinar em Pesquisa, Universidade São Francisco, Bragança Paulista 12916-900, Brazil; juliana.sciani@usf.edu.br

**Keywords:** toxins, venoms, skin secretion, drug discovery

## Abstract

Among the vast repertoire of animal toxins and venoms selected by nature and evolution, mankind opted to devote its scientific attention—during the last century—to a restricted group of animals, leaving a myriad of toxic creatures aside. There are several underlying and justifiable reasons for this, which include dealing with the public health problems caused by envenoming by such animals. However, these studies became saturated and gave rise to a whole group of animals that become neglected regarding their venoms and secretions. This repertoire of unexplored toxins and venoms bears biotechnological potential, including the development of new technologies, therapeutic agents and diagnostic tools and must, therefore, be assessed. In this review, we will approach such topics through an interconnected historical and scientific perspective that will bring up the major discoveries and innovations in toxinology, achieved by researchers from the Butantan Institute and others, and describe some of the major research outcomes from the study of these neglected animals.

## 1. Introduction

**Figure 1:**
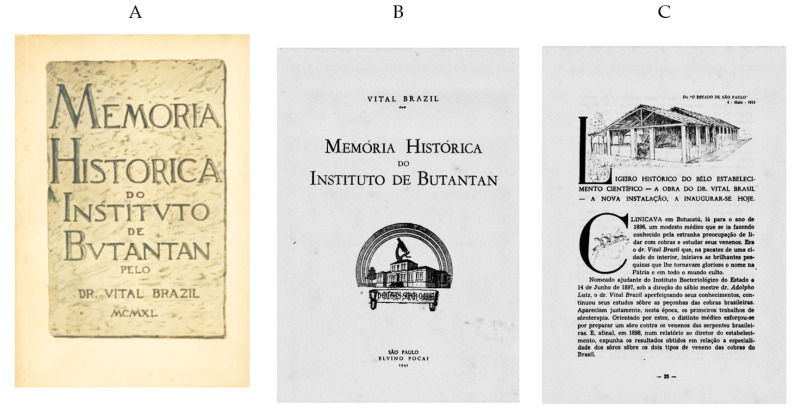
Selected reproductions of (**A**) the book written by Vital Brazil in 1941. This and other classic books are available at https://bibliotecadigital.butantan.gov.br/, accessed on 19 November 2021. Please note the institute logo in (**B**). It is the depiction of the main-laboratory building, underneath a microscope, bearing the motto “peritas super omnia”, meaning “expert in everything” in Latin. (**C**) News advertising the inauguration of new facilities at the Institute, in 1914.

“Around 1896, a modest physician that used to practice medicine in Botucatu became notorious due to his strange fascination with snakes and their venoms. It was Dr Vital Brazil that, from the tranquility of the countryside, was taking the initial steps on the brilliant research that would make him famous not only in Brazil but also all over the educated world”. This free translation of the beginning of the first paragraph (Figure 1C) of the book “Memória Histórica do Instituto Butantan” (Figure 1A; Historic memory of Butantan Institute, in free translation) written by Dr Vital Brazil himself (Figure 1B) [1] refers to published news in 1914 reporting the inauguration of ‘new facilities’ in the Institute (Figure 1C).

**Figure 2:**
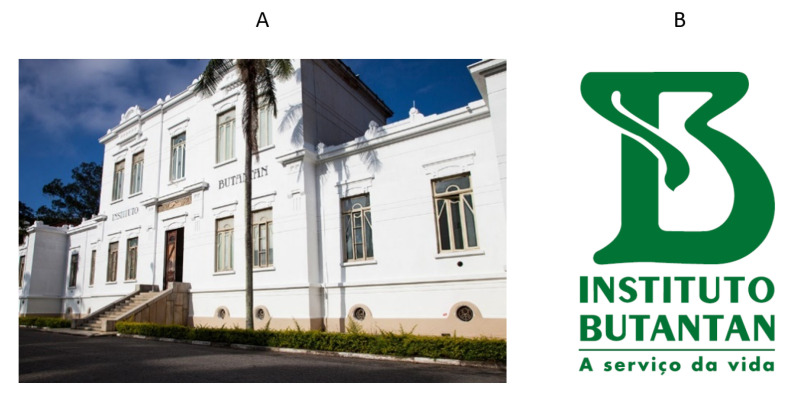
(**A**) A recent photograph of the main-laboratory building, in a similar framing as depicted in Figure 1. (**B**) Current Institute logotype, bearing the slogan ‘A serviço da vida’ (at the service of life, in free translation).

Over one hundred years after the news reported above, some of the authors of this review have worked, conducted research, performed experiments, and published papers in this exact same building. Since then, a lot has changed in the Institute, including its slogan, but not the building (Figure 2). Our slogan is now “at the service of life”, a humbler commitment to the institutional mission, and the research laboratories have been decommissioned from this building, which is currently undergoing restoration and will be dedicated to cultural activities only.

### 1.1. The Origins of Negligence

There are twenty (tropical) diseases that are officially classified as ‘neglected tropical diseases’ by the World Health Organization (WHO) [2]. Neglected tropical diseases persist under conditions of poverty and are concentrated almost exclusively in impoverished populations in the developing world. They are: Buruli ulcer, Chagas disease, Dengue and Chikungunya (only WHO, not CDC), Dracunculiasis, Echinococcosis, Yaws, Fascioliasis, African trypanosomiasis, Leishmaniasis, Leprosy, Lymphatic filariasis, Onchocerciasis, Rabies, Schistosomiasis, Soil-transmitted helminthiasis, Cysticercosis, Trachoma, Scabies and other ectoparasites, Snakebite envenoming, Mycetoma and deep mycoses. These diseases are common in 149 countries, affecting more than 1.4 billion people (including more than 500 million children) and costing developing economies billions of dollars every year.

The importance of neglected tropical diseases has been underestimated since many are asymptomatic and have long incubation periods. The connection between a death and a neglected tropical disease that has been latent for a long period of time is not often realized. Additionally, neglected tropical diseases are often associated with some kind of social stigma, making their treatment more complex.

From the toxinology perspective, one can also consider that there are ‘neglected’ venomous and poisonous animals by employing very similar criteria to justify such negligence: Human accidents occur with individuals who are often amongst the poorest populations, living in remote, rural areas, urban slums or conflict zones; the accident causes no rapid death of the victim and/or such animals are stigmatized (cause bad luck, carry evil spells or are cursed).

Depending on the nature and origin of the venom or toxin, one can clearly perceive that there are ‘preferred’ subjects and matters in the field of toxinology (Table 1). Probably due to historical and/or epidemiological factors, some animals and venoms—normally the ones that elicit acute, severe lesions due to some pronounced biological activity—were selected (or elected) as ‘more relevant’ to the field and have been thoroughly studied throughout the years. Endemic animals, such as spiders and scorpions that have adapted to urban environments, have also ‘deserved’ more attention than other species. All the consulted databases indicated that there is more literature on snakes, spiders and scorpions (the triad) than the others. Interestingly, Scopus and Web of Science present the same publications ratio for triad:neglected (7.8), whereas Google and PubMed display lower ratios (5.5 and 1.8 respectively), probably due to the differences in indexed publications queried.

The aim of this review is, therefore, to shed a light upon such amazing animals and their venoms and secretions, presenting a non-anthropocentric view of their venom composition and the (few, but consistent) biomedical ‘cases’ derived from the study of such species, and review the literature and the biotechnological developments derived from venoms and secretions from toxic animals that have not received proper attention from the scientific community over the past years and cast a light on their unique features and interesting molecules. Afterall, just like the neglected tropical diseases, it was never about the ‘importance’ of these animals, only their ‘relevance’, i.e., their economic impact, geopolitical localization, affected population, endangerment status and profit potential, in addition to formerly listed reasons.

### 1.2. Biodiversity

Earth’s existing biodiversity is a direct consequence of Darwin’s Natural Selection, i.e., the survival of the fittest, in a constant struggle to survive [3]. With an estimated 8.7 million species inhabiting our planet, the mere 1.2 million (mostly insects) that have already been identified and described have all—or are still in the process of—adapted and evolved so that, after numerous breeding cycles, poorly suited individuals are filtered out by nature.

One particularly interesting adaptation which emerged millions of years ago was the biochemical weaponry utilized for defense and/or predation by some organisms as a means of guaranteeing survival [4]. These so called ‘toxins’ can be found in procaryotic species, such as *Staphylococcus aureus* and *Klebsiella pneumoniae* [5,6], plants (*Cicuta maculate* (Socrates committed suicide by drinking cicuta, circa 399 B.C.) and *Nicotiana tabacum* (homage to Jean Nicot de Villemain, who introduced snuff to the French court in 1560)) and, obviously, animals.

For animals, these toxins are believed to have originated from ancestral house-keeping genes that underwent variation and neofunctionalization [4,7], resulting in molecules displaying an ‘increased’ biological activity, normally targeted to major biological systems that when unbalanced may result in severe risk of death, such as the hemostatic-interfering molecules. The toxins were then specifically expressed in venom-secreting cells that eventually became specialized venom glands [8]. Such specialization became an evolutionary advantage, due to unique pharmacokinetic properties that these (typically) peptides and proteins granted to such animals [9,10].

### 1.3. Toxins: Snakes, Spiders and Scorpions as Classical as It Can Be

Toxinology has its origins long associated with venomous animals and not poisonous ones. There might be some controversy in this separation, but it is commonly accepted that venomous animals would possess an inoculating apparatus capable of delivering toxins into the prey/aggressor. On the other hand, poisonous animals would secrete toxins in their skin or body organs and would have to be actively eaten/beaten/attacked/poked/colonized (bacteria) in order for to the toxins exert their effect.

Nevertheless, mystical, magical, medical and/or lethal uses of some animals’ venoms are well known throughout history. For example: Cleopatra may have committed suicide by letting herself be bitten by a snake (*Naja haje* probably). In the Bible there are nine verses citing scorpions (Luke 10:19 and 11:12, Kings 12:11 and 12:14, Deuteronomy 8:15, Ezekiel 2:6, Revelation 9:3, 9:5 and 9:10). Greek mythology presents us the Lernaean Hydra, a serpentine water monster with many heads (depending on the myth source) with poisonous breath and blood so virulent that even its scent was deadly, as well as the Medusa, one of the three monstrous Gorgons, generally described as winged human females with living venomous snakes in place of hair.

These venomous animals are still present in modern-day fiction, such as the famous Spiderman, whose superpowers derived from mutations resulting from the bite of a radioactive spider. Even Harry Potter was forced to deal with the Basilisk, a giant snake capable of instant kill just by gazing at the victim’s eyes. There are also urban legends and local habits, such as the well-known North American arachnophobia.

On the other hand, poisonous animals share a less glamorous role in human history. They have participated, for example, in human (sacrificial) rituals and attempted pharmaceutical developments throughout history. There were Maya human bloodletting rituals that employed the sting of marine stingrays as blades, due to a ‘more efficient’ bleeding [11]. Hunters have long sought the Central and South American Dendrobatidae ‘poison arrow frogs’ (self-explanatory) to use their toxic skin secretion for hunting [12]. Traditional Chinese medicine uses the ‘all healing’ Chan’Su (dried *Bufo bufo* skin) for mostly any illness [13]. Amazon tribes traditionally used Kambo (or Kampum) in their purification rituals [14]. This medicine is extracted from *Phyllomedusa bicolor* skin secretion and has become known as the ‘frog vaccine’ in urban environments. The Bible also cites such animals in the infamous passage in Exodus 8:1–4, in which the “*great LORD says: Let my people go, so that they may worship me. If you refuse to let them go, I will plague your whole country with frogs. The Nile will teem with frogs. They will come up into your palace and your bedroom and onto your bed, into the houses of your officials and on your people, and into your ovens and kneading troughs. The frogs will go up on you and your people and all your officials*”. Unfortunately, the poisonous animals are presented from a more neglected, less charming perspective, as presented above.

All this glamour associated with venomous animals has led to the establishment of what can be considered the ‘greatest-hits’ of (classical) toxinology: snakes, spiders, and scorpions (the triad). Undoubtedly, studying these animals’ venoms has yielded a myriad of relevant scientific papers [15,16,17,18,19] produced by highly committed international scientific groups. The molecular dissection of the venom constituents has made it possible that effective sera could be manufactured [20,21,22], thus reducing mortality and morbidity associated with envenomation [23,24]. Moreover, one of the world’s most administered antihypertensives (Captopril) is a direct derivative of one viper toxin [25].

Another example is a tumor-labeling molecule (tozuleristide), currently undergoing clinical phase 1 studies, that is being used in surgeries as marker and diagnostics for glioma and other tumors. This molecule is an analogue of a chlorotoxin isolated from the venom of the scorpion *Leiurus quinquestriatus* [26,27,28].

It is noteworthy to mention that there is young blood trying to join the party. Even though the marine mollusks of the *Conus* genus do not belong to the classic triad, they are becoming more and more famous since the discovery of ziconotide (Prialt), the strongest analgesic ever described: a calcium channel blocker, purified from the *Conus magnus* venom [29]. These animals are discussed below.

However, even for such well-studied animals there are still ‘neglected’ molecules present in their venoms, such as L-amino acid oxidase, crotapotin, crotamine that ‘simply’ for not killing or harming the animal models are put aside, turning the spotlight to the super-toxic metallopeptidases, phospholipases (A_2_ and D) and ionic channel blockers.

Still, a number of other animals can (and do) cause accidents upon human encounters, displaying broad variation in terms of the clinical outcome. Marine animals are good examples: sea urchins can be solely painful [30] whereas mollusks can instantly kill [31]. Yet, for some reason, such animals have not been able to attract the attention of major research groups in toxinology, remaining in ‘neglect’ for the past couple of decades.

The modern reptiles are a group comprised of the Crocodila, Lepidossaura, Rhynohocephalia, Squamata, Testudines and Aves. With the exception of snakes, no other true venomous reptile (i.e., with a specialized venom inoculation apparatus) is currently known. The venomous living dinosaurs, i.e., birds pitohui, ifrita and rufous [32], and the Komodo dragon are considered to be poisonous [33,34].

**Figure 3:**
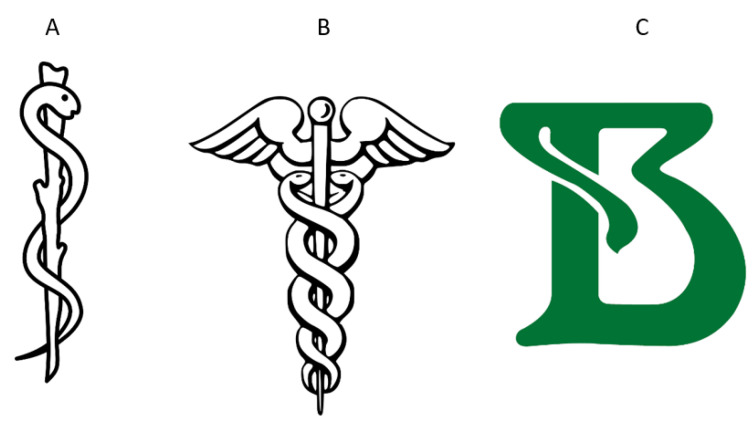
(**A**) Rod of Asclepius, (**B**) the caduceus and (**C**) Butantan Institute logotype.

However, in the end, snakes are the most classical venomous animals. Since ancient times, their behavior has been considered to be mischievous—even tempting—and their venom has been associated to magic spells and even cures. Not surprisingly, The Rod of Asclepius, i.e., the Medicine symbol (Figure 3A), is a snake serpentizing around a rod [35]. Nevertheless, the caduceus—the traditional symbol of Hermes—represented by two snakes serpentizing around a winged rod (Figure 3B) is often mistakenly used as a symbol of medicine instead of the Rod of Asclepius, especially in the United States, as a consequence of documented mistakes, misunderstandings and confusion in the late 19th and early 20th centuries. However, the two-snake caduceus design has ancient and consistent associations with trade, eloquence, negotiation, alchemy, and wisdom. Last, but not least, the current Butantan Institute logotype (created in 1983) is a clever design in which the capital ‘I’ and ‘B’ are fused and the ‘B’ serif becomes the snake serpentizing around the ‘I’, which serves as the rod (Figure 3C).

Jumping a few centuries ahead, there is indeed current medicine based on snake venoms, such as Captopril [25,36,37], Aggrastat, Intergillin and Aggretin [30,33], proving that ancient wisdom may be old, but never outdated. Not only that, but this particular *Toxins* issue that celebrates the 120th anniversary of Butantan corroborates this. At the same time, one can easily note the iconic fascination that the snake has exerted over the local scientific community, that has—and still does—followed Vital Brazil’s initial steps.

### 1.4. Lizards

Lizards’ biting has long been discussed among the toxinology field due to the lack of an inoculating venom apparatus. *Heloderma* bites have been reported since 1882 [38,39], and the first toxic activities were described in 1900–1950. At that time, authors were aware that such lizards’ toxins included neurotoxins, causing respiratory depression. Inflammation, edema and pain have also been described. However, once this animal bites ‘as strong as a bulldog’ according to the authors, these symptoms may not be exclusively toxin-derived [40]. Moreover, its hemolytic activity is mild, when compared to snakes, and seems to be species-specific [41].

Later, between 1950–1990, a wide range of biological activities were described, such as phospholipasic, hyaluronidasic, proteolytic [42], L-amino acid oxidase, fibrinolytic, [43] esterase, 5’-nucleotidase [44], secretagogue [45] and nerve growth factor activity [46]. Furthermore, new venom components (at the time) were isolated and identified, such as: kallikrein [47,48], Helospectins 1–2 (acting as secretagogues) [49], Gilatoxin (serine peptidase) [50], Helodermin (vasoactive peptide) [51], and Helothermine (CRISP) [52]. Hyaluronidase [53], a Phospholipase A2 [54], Helodermatin (hypotensive toxin) [55], and Exendin-3 (secretagogue) [56] were also described. Such myriad of toxins could, then, be correlated to the many established envenomation symptoms, such as hypotension and respiratory difficulties [57], smooth muscle contraction [58] and anticoagulant effect [59].

In 1992, Exendin-4 identification was a major event and *Heloderma* venom studies skyrocketed from this year onwards [60]. Several research projects have evaluated the antidiabetic potential of this molecule, which gave rise to exenatide, a new drug for the treatment of diabetes [61]. A few years later, the inhibition of platelet aggregation by a phospholipase isolated from a Helodermatid lizard was described [62]. Even though it was already known that *Heloderma* venom presents at least five anionic phospholipases, being the most abundant similar to *Apis mellifera* phospholipase [63], it was another important event.

In the following years, Helokinestatin, a toxin that acts as an antagonist of the bradykinin B2 receptor, was described [64]. Moreover, Helofensin was identified by Fry and co-workers by genetic and functional analysis [65], and classified together with a class of lethal toxins firstly described by Komori at al. in 1988 [66]. The presence of a natriuretic peptide in *Heloderma* venom was pointed out by different authors [67,68].

A work comparing the venom proteome of *Heloderma suspectum* with the venom of *H. exasperatum* and *H. horridum* presented an interesting result. Although *H. suspectum* was evolutionarily separated from the other species 30 million before, the venom composition was basically the same for the three species, presenting the same toxins with slight differences in their relative proportions [69]. Moreover, authors could also describe two new molecules: semaphorin and a bactericidal/permeability-increasing (BPI) molecule. Another study that characterized the *H. suspectum* venom proteome relates to the presence of a neuroendocrine convertase 1 homolog, and proposes that this protein is responsible for the cleavage of the proforms of exendins. In the same study, the authors also point out the high presence of phospholipase propeptides in the venom proteome [70]. Recent works allowed access to different classes of proteins, and also new biological activities from *Heloderma* venom. The venom gland transcriptome analysis from *H. horridum horridum* revealed the presence of metalloproteases, lipases, vespryns, waspryns, lectins, cystatins and serine peptidase inhibitors, but none of these proteins were actually isolated from the venom [71]. Furthermore, *Heloderma* contains neurotoxins in its venom, and these toxins are able to bind sodium and calcium channels [72]. An important work by Fry et al. [73] evaluated phylogeny between snakes and lizards and demonstrated that the venom delivery system of these animals could have evolved from the same common ancestor. This was the first study that biochemically evaluated the venom of a lizard from the Varanidae family. The crude venom from *Varanus varius* displays a hypotensive effect and an isolated PLA2 from the venom inhibits platelet aggregation, via adrenaline pathway. The LC-MS analysis indicates the presence of natriuretic peptide, PLA2, CRISP, and Kallikrein. cDNA libraries analyses indicated the presence of AVIT, cobra venom factor, cystatin, crotamine, nerve growth factor and vespryn. Later studies demonstrated that the venom of *V. komodensis* (Komodo Dragon) also induces a hypotensive action, and that the venom is composed of toxins, such as PLA2, kallikrein, natriuretic peptide, CRISP, and AVIT [74].

A cDNA libraries analysis conducted by Fry et al. [67], comparing different lizards, was able to reveal new classes of toxins presents in the Varanidae family, such as lectin, veificolin, hyaluronidase, Cholecystotoxin (binds to CCK-A), Celestoxin (hypotensive), epididymal secretory protein and Goannatyrotoxin (hypertensive/hypotensive effect).

Then, the venoms of *Lanthanotus*, *Varanus* and *Heloderma* genus were compared through proteomic approaches and enzymatic activities profiling [69]. Interestingly, the only ubiquitous protein was Kallikrein and, different from Heloderma, which presents a conservation of venom constitution and actions in different species, the Varanus genus presents a variability in venom proteins and enzymatic activities such as serine peptidases, phospholipase activity and differential potential to cleavage alpha and beta chains from fibrinogen.

Venoms from different species of the Varanus genus were evaluated for the ability to prevent blood clotting by thromboelastography, and the venoms differ regarding the activity; the most potent effects were found in arboricole species, probably due to the selective pressure, according to the authors [75]. Similar to *Heloderma*, Varanid lizards possess neurotoxins that are able to bind sodium and calcium channels [72].

### 1.5. Amphibian

Although a witch’s recipe benefits from venomous animals, the toe of a frog and the eye of a newt would definitely spice things up. Shakespeare’s Macbeth (Act 4, Scene 1) contains a recipe for a witch’s brew that goes as follows:

“Fillet of a fenny snake,

In the cauldron boil and bake;

Eye of newt and toe of frog,

Wool of bat and tongue of dog,

Adder’s fork and blind-worm’s sting,

Lizard’s leg and owlet’s wing,

For a charm of powerful trouble,

Like a hell-broth boil and bubble.”

Although most of the above referred ingredients can be traced back to herbs (eye of newt = mustard seed (*Sinapis alba*); toe of frog = buttercup (*Ranunculus acris* L.); wool of bat = holly leaves (*Ilex aquifolium*); tongue of dog = gypsyflower from the genus hound’s tounge (*Cynoglossum officinale* L.); adder’s fork = least adder’s-tongue (*Ophioglossum lusitanicum* L.); blind-worm = slowworm (*Anguis fragilis*)), a really mighty witch might as well as go on literally, seeing the true herpetological powers needed for the spell.

According to Table 1, published papers on amphibian venoms are less common than the triad. The similar figure to scorpion papers is due to two characteristics belonging to the study of the amphibian skin secretion: (i) the discovery of magainin, the first antibiotic peptide by [76], which boosted the literature by making several researchers seek other antibiotic peptides in other species, and (ii) the vast Chinese literature on Chan’Su, the all healing Chinese traditional medicine. These two events have undoubtfully contributed to these numbers. However, in general, amphibian literature on accidents is scarce in comparison to venomous animals.

The amphibian defense strategy against predators/aggressors is the “passive” defense (with the exception of *Rhaebo guttatus*, which is capable of voluntarily compressing its parotoid glands and ejecting its contents [77]), and the chemical nature of their venom is mainly protein/peptide toxins and low-molecular-mass compounds (such as alkaloids, steroids and their respective derivatives).

Some of the authors of the current review have been working with amphibian skin secretion for almost twenty years. As a consequence, they have been able to produce consistent literature on the subject that encompasses the different classes of bioactive molecules commonly found on the amphibian skin secretion. A compilation of these results will be presented below, together with the related literature.

Conceição, et al. [78] have evaluated the skin secretion of the tree frog *Phyllomedusa hypochondrialis* and described that this secretion presents proteins ranging between 68 and 14 kDa, and that proteolytic and phospholipase A2 activities could be detected in vitro. Moreover, authors also report that the injection of 0.6 ug of the venom in mice induced myotoxicity, as evaluated by the increase of creatinine-kinase activity in plasma. The same dose of the skin secretion also elicited vasoconstriction (for 20 min) and leukocyte rolling, as assayed by intravital microscopy. Edema and nociception, in a dose–response manner, could also be observed. Interestingly, the observed vascular permeability alterations displayed a different mechanism, in which the lowest tested concentration caused the most intense effect, in comparison to larger concentrations. This phenomenon is mostly likely due to the presence of different molecules, in distinct relative concentrations, acting on independent biological systems.

As a consequence of the described leukocyte rolling effect, a subsequent study was performed [79] that assessed the mechanisms involved in that effect. Experiments revealed that the toxins could lead to edema formation, within 2 h, which lasted for 24 h. Moreover, authors also described that the numbers of rolling and adherent leukocytes were augmented in post-capillary venules. Cytological analysis showed that macrophages were the main cells present 2 h after the injection, whereas neutrophils were the cells present after 24 h. The cytokine profiles indicted elevated levels of chemokines MCP-1 and KC, and also IL-6 and PGE2.

Mendes et al., 2016 [80], studied the casque-headed tree frog *Corythomantis greening*, a frog bearing a cranial bone adaptation used in phragmosis. The cutaneous secretion of this animal was able to induce inflammation (edema, for 96 h after the injection) and nociception. Moreover, relevant enzymatic activities were detected in the skin secretion, such as fibrinogenolytic, hyaluronidasic and metallopeptidasic. Enzymes presenting such activities have already been described as important toxins for snake venoms [81,82] and were also described in some amphibians from different genus, for example phospholipase in *Pithecopus azureus* [83] and serine peptidases in *Duttaphrynus melanostictus* [84]. Furthermore, Fusco et al. 2020 [85] studied the epidermal secretion of *Argenteohyla siemersi* and described both phospholipasic and hemolytic activities. They also reported that that venom is cytotoxic and capable of promoting necrosis which is independent of the proteolytic activity, a different activity pattern from *C. greeningi* (included in the same genus).

Targeting antibiotic peptides—a consequence of Zasloff’s study—Conceição et al., 2006 [86], screened the skin secretion of *P. hypochondrialis* for antimicrobial peptides against Gram-positive and -negative bacteria and successfully described Phylloseptin-7 and Dermaseptin (DPh-1). These peptides were active over common pathogens, such as *Staphylococcus aureus*, *Escherichia coli*, *Pseudomonas aeruginosa* and *Micrococcus luteus*. In a complementary study, Huang and collaborators identified a new Dermaseptin from the same *P. hypochondrialis* (Dermaseptin-PH), which was active against Gram-positive/-negative bacteria and inhibited biofilm formation. This peptide was also effective against *Candida albicans.*

Other authors also reported complementary phylloseptins. For example, Wu et al., 2017 [87], isolated PNS-PC from *P. camba* the PNS-PC. This peptide displays inhibitory action against Methicillin-resistant *Staphylococcus aureus*. They also isolated PBa1–3 from *P. Burmeister*, a peptide with antibacterial and antifungal activities [88]. A recent study by Liu et al., 2020 [89], reported the antibacterial activity of PV-1, a Phylloseptin from *P. vaillantii* in vitro and in vivo. In spite of observed hemolysis (in vitro), this peptide was not toxic to hepatic and renal tissues in vivo, indicating the possible therapeutical potential of this peptide for bacterial infection.

Zhang et al., 2010 [90], isolated Phylloseptin-1 (PSN-1) from *P. sauvagei*. This peptide was active against *Staphylococcus aureus* in vitro, including bacterial biofilm formation inhibition. A few years later, Raja et al., 2013 [91], described five more Phylloseptins displaying antimicrobial activity from this species. Their work proved that the structural differences among those peptides were responsible for the different observed bactericidal potency, suggesting that the alpha-helix amphipathic conformation leads to microbial membrane disruption.

Using Zasloff’s classic strategy, Conlon et al. 2007 [92] stimulated *Hylomantis lemur* skin secretion with norepinephrine and successfully purified Dermaseptin-L1 and Phylloseptin-L1, which were active against Gram-negative bacteria and *Batrachochytrium dendrobatidis*, a fungus that infect frogs.

In 2009, an unexpected antimicrobial peptide was described by Sousa et al. [93]. Leptoglycin, a peptide comprised basically by Leu and Gly (with an import Pro at the center of the sequence) was isolated from the skin secretion of *Leptodactylus pentadactylus* and was active against Gram-negative bacteria.

Bradykinin-potentiating peptides are protagonists of the most important example of drug discovery from animal venoms. Rocha e Silva’s discovery of bradykinin [94] ultimately led to the discovery of the bradykinin-potentiating peptides (BBPs) from snake venoms. Such a peptide, on the other hand, led to the development of Captopril, the first drug belonging to angiotensin-converting enzyme inhibitor (ACEi) class, widely used around the world to treat arterial hypertension. In another unexpected study, Conceição et al., 2007 [78], described the first canonical BPP isolated from another source than snake venoms. Phypo-Xa, a decapeptide isolated from *P. hypochondrialis,* inhibited ACE and potentiated bradykinin both in vivo and in vitro. A few years later, those authors [95] also isolated three bradykinin-related peptides from *P. nordestina* skin secretion: two were vasodilators (Pnor3 and Pnor7) and one was a vasoconstrictor (Pnor5).

Some amphibians, particularly toads, can be considered major biological sources of low-molecular-mass compounds, such as alkaloids and steroids. Tempone et al. [96], through biomonitored assays, have isolated two bufadienolidc steroids displaying antiparasitic activity from the skin secretion of *Rhinalla jimi*. Telecinobufagin and hellebrigenin were not new molecules at that time; however, the activity against *Leishmania* sp. promastigotes and amastigotes in macrophage culture (without NO production modulation) and the anti-*Trypanossoma cruzi* trypomastigotes activity were the novelties they reported in their paper. The mechanism of action of these molecules seems to be related to the disturbance of cellular membrane and mitochondrial function. Neither steroid presented hemolytic or cytotoxic activities in the tested conditions.

That same group of authors [97] later assayed the skin secretion of *P. nordestina* on antiparasitic models. They were able to demonstrate that four antimicrobial peptides (Dermaseptins 1 and 4, and Phylloseptins 7 and 8) were able to decrease the in vitro viability of *T. cruzi*, with a high theoretical therapeutic index. The proposed mechanism of action of the peptides is cell death induction, through cellular membrane permeabilization. Phylloseptin-7 was also effective against *Leishmania* sp.

Such results (selective membrane permeation) convinced Sciani et al. [98] to investigate the possible antitumor activities of the skin secretion of some Brazilian toads. MCF-7 and MDA-MB-231 lineages (breast tumor) displayed reduced proliferation and apoptosis induction when treated with eight different amphibian skin secretions. Among them, the most promising results came from *R. guttatus*, *R. margaritifera* and *P. hypochondrialis*. Moreover, *R. guttatus* and *R. marina* displayed selective antitumor activity over HL-60 (leukemia lineage), without toxicity to human leukocytes. It is believed that the observed antiproliferative effect is due to the known presence of bufadienolides in this toad secretion.

Schemda-Hirschmann in 2014 [99] related the presence of argininyl bufadienolides in *R. schneideri* dermic secretions, which were active on different tumor lineages AGS, SK-MES-1, J82 and HL-60 (gastric adenocarcinoma, lung carcinoma, bladder carcinoma and leukemia, respectively). Later, the same group showed similar activity in the Peruvian *R. marina* venom, and the mechanism of action seems related to ROS production and cell cycle arrest, for breast cancer lineages [100]. Antitumor properties were also described for the Paraguayan *Rhinella* sp. Such skin secretion is traditionally used by locals in folk medicine to treat skin lesions and tumors [101].

The crude extract of *Physalaemus nattereri* is cytotoxic for the B16F10 melanoma cell line. Carvalho et al. [102] observed that the secretion was able to induce conformational changes in cells, exposure of phosphatidylserine on cell membrane, reduction of mitochondrial membrane potential and arrest of cell cycle in S phase, indicating that apoptosis is the probable mechanism of action that explains the antitumor activity. RP-HPLC fractionated *P. nattereri* extract points out that this biological action is due to peptides

Skin venom from the Malaysian toad *B. asper* was active against HCT 116 colorectal tumor line by apoptosis induction, via caspase 3/7 activation and mitochondrial membrane potential disruption [103]. Bufadienolides also possess the ability to inhibit Na^+^/K^+^ ATPase and trigger caspase-induced apoptosis, being more selective to cancer cells than normal cells [104]. The venoms of two Turkish Salamandrine amphibians were tested against cancer cell lineages. The venoms, which presented proteins in their biochemical content, were active against cervix, alveolar, colon colorectal, pancreas, prostate, astrocytoma and breast carcinoma lines. However, these secretions were also toxic to human fibroblasts (HEK 293) [105].

Marinobufagin is a molecule present on *R. marina* venom displaying activity against leukemic cells without being toxic to normal blood cells. According to Machado et al. [106], this steroid induces toxicity via apoptosis, antimitotic action and cycle cell arrest at interphase in leukemia cells, without any genotoxicity.

The bufadienolides, bufotoxins, alkaloids and arginiyl derivatives from *R. jimi* cytotoxicity effects on cancer cell lineages were studied by Filho et al. [107], whereas Spinelli et al. [108] revaluated the antitumor action of 11 different Argentine amphibians: 6 *Hylidae*/*Microhylidae* and 5 *Leptodactylidae*. These venoms induced apoptosis and autophagy. Interestingly, *Leptodactylidae* skin secretion induced aggregation on cancer cells.

Finally, we present bufotenine: a tryptamine alkaloid found in many species and genera across nature (animals and plants), particularly in *R. crucifer*, *R. granulosa*, *R. schneideri*, *R. icteria* and *R. jimi* [109]. This molecule was selected in biomonitored assays and has the capacity to inhibit the penetration of rabies virus in mammalian cells, through an apparent competitive mechanism [110]. Complementary studies conducted by those authors [111] showed that this molecule was active in vivo, by increasing the survival rate of intracerebrally virus-infected mice from 15 to 40%. The safety of bufotenine was then evaluated [112] and no significant effects on mice could be detected at the effective antiviral dose. Interestingly, bufotenine acts synergically with ocellatin-F1—an antimicrobial peptide obtained from the frog *Leptodactylus labyrinthicus* skin secretion—in the rabies virus model [113]. Finally, recent in vitro assays showed that bufotenine has no antiviral action against canine coronavirus (CCoV), canine adenovirus type 2 (CAV-2) or herpesvirus type 1 (HSV-1), indicating some specificity against distinct types of viruses [114]. The mechanism of action of this alkaloid remains unclear (although the evaluation of its effects in the immune system is being assayed by these authors), but bufotenine is the perfect example of the potential of bioactive molecules isolated from a neglected venom, serving as biotechnological tool for a neglected disease drug development study.

### 1.6. Marine Animals

Oceans dominate planet Earth: approximately 70% of the Earth’s surface is covered by water, and from that, 96.5% of this water is from the oceans [115]. More than 480,000 species of marine animals have been discovered and identified according to the World Register of Marine Species [116]. However, such a figure may be even larger: the Ward Appeltans of the Intergovernmental Oceanographic Commission of UNESCO (https://en.unesco.org/news/ocean-life-marine-age-discovery-0, accessed 19 November 2021) estimates that oceans may hold 700,000 species. These data represent what the ocean can become: a molecular library! Molecules that belong to an organism’s physiology, act on hunting and prey digestion and/or chemical defense may ultimately lead to the discovery of new compounds with biotechnological or pharmaceutical uses.

Regarding bioprospection, marine animals have provided several molecules for a wide range of therapeutic applications. Some of them have already been approved by regulatory agencies and are being commercialized. The most known is ziconotide (Prialt), a ω-conotoxin peptide from *Conus magus*, applied by intrathecal route as analgesic for chronic and intense pain, whose mechanism of action is the selective blocking of neuronal N-type voltage-sensitive calcium channels [117,118]. Another known drug from marine animals is trabectedin (Yondelis), initially isolated from the marine ascidian *Ecteinascidia turbinata*, used to treat sarcomas and ovary cancer [119].

For cancer, other drugs have been developed, such as Ara-C (Cytarabine), a nucleoside isolated from a Caribbean sponge, *Cryptotheca crypta*. It is used for certain types of leukemia, including acute myeloid leukemia, acute lymphocytic leukemia and chronic myelogenous leukemia [120].

Brazilian sponges and cnidarians, such as *Zoanthus sociatus*, *Exaiptasia pallida* and *Carijoa riisei*, have yielded promising molecules active on cancer cells. Some of these authors have showed that *C. riisei* and the porifera *Tedania brasiliensis* extracts were effective in reducing the cell viability of glioblastoma, and that *C. riseii* also acts on breast and ovary cancer. Moreover, *Z. sociatus* and *E. pallida* were able to diminish leukemic cell viability [121]. Regarding the envenomation field, some of these authors have contributed for the understanding of marine animal venoms, from a biochemical and pathophysiological perspective.

### 1.7. Sea Urchins

Sea urchins are the most abundant animals in Brazilian shores. They are also responsible for the majority of reported marine animal accidents [122]. *Echinometra lucunter*—the rock boring urchin—can be easily found in rocky shores. Human accidents are frequent and can be associated with the animal’s manipulation by bathers, or by people stepping on the animals while walking on the shore. More severe cases (in terms of the number of spines punctures) can result from people being dragged onto rocky walls by wave action. Still, the most common route that the spines penetrate the skin is through the foot or hand. This event causes local inflammatory reactions, characterized by edema, erythema and pain [123,124]. Facing this problem, the authors have wondered: is this accident solely mechanical due to the spine’s penetration, or does the sea urchin have a venom that contributes to the described symptoms?

To answer that question, ‘toxins’ from *E. lucunter* spines were extracted, immersing the excised appendices in a physiological buffer (to avoid cell lyses by osmotic shock), followed by animal inflammation test models. Authors described that the extract induces a pro-inflammatory reaction, by increasing rolling, adhered and migrated leukocytes. Moreover, the spines extract decreased the pain threshold and induced paw edema [30]. In another study, these authors were able to isolate one molecule responsible for those effects, including its partial molecular characterization [125]. However, it was clear that there was more than one single molecule eliciting such activities; therefore, the clinical observed symptoms clearly surpass the mechanical trauma aroused by spine penetration.

This mechanism is a very successful adaptation: the venom (i.e., the ‘toxins’) diminishes the pain threshold—making the victim more susceptible to painful stimuli—at the same time that the spines puncture the skin. As a consequence, the mechanical accident becomes more aggressive, due to this synergism (resulting in inflammation).

*E. lucunter* spines do not contain typical venom glands, in the same way venomous animals do, but it is a living structure, full of granular cells, which are most likely to produce and secrete these toxins along the entire spine, particularly at in the spine tip, a region more susceptible to mechanical stress by contact (with possible predators and aggressors) [126]. Moreover, although the spine is composed mainly of calcium and/or magnesium carbonate, the myriad of cells embedded would significantly contribute to spine regeneration. It has been demonstrated that the spine secretes cathepsins B and/or X, an enzyme associated with matrix remodeling processes, contributing to the spine growth and regeneration, but also to the toxicity.

Besides spines accidents, consumption of sea urchins may elicit undesirable/toxic effects for the consumer, as they are usually eaten raw. Therefore, these authors have investigated the coelomic fluid of *E. lucunter*, searching for toxins (pro-inflammatory molecules, in particular). A bioactive peptide, termed ‘echinometrin’, capable of reducing rolling cells and increasing adhered and migrated ones—concomitant to edema induction—was identified. Moreover, this peptide induced mast cell degranulation, which makes us think that histamine was responsible for the observed inflammatory reaction [127]. Actually, many consumers present allergies after the consumption of raw sea urchin, and there are studies suggesting the participation of vitellogenin in such process, by increasing IgE levels [128,129]. Echinometrin is, in fact, a cryptide [130], i.e., an internal fragment of vitellogenin. Moreover, its N- and C-termini match the amino acid specificity for (the previously reported) cathepsin B/X, suggesting a local toxin generation system, in which both substrate and processing enzyme are present and ready to act.

Once the biomonitored assay reported above proved successful in the identification of one bioactive peptide, these authors decided to performed an untargeted peptidomic approach on sea urchins’ peptides. The secreted peptides from *E. lucunter*, *Lytechinus variegatus* and *Arbacia lixula* were analyzed. It was possible to observe that coelomic fluids of all three species are full of peptides. On the other hand, peptides could be identified only in the spines of *L. variegatus* and *A. lixula*, whereas *E. lucunter* spines contain mainly low molecular mass compounds. Database mining suggests that some peptides may display relevant biological effects, such as antibiotic, anticancer, antiviral, phospholipase A2 inhibitor and neuroprotective properties, making sea urchin molecules a source of new therapeutic compounds [131].

### 1.8. Mollusks

Peptides are abundant in marine mollusks from the Gastropoda class. They are usually referred as ‘conopeptides’ and are responsible for prey paralysis due to their specific action on the neuromuscular ionic channels [132,133]. The genus *Conus* is a well-known source of these conopeptides. The Tox-Prot database from Uniprot/Swiss-Prot describes that 1.370 toxins are manually annotated for 117 snail species, most of them from genus *Conus* [134,135]. On the other hand, the database platform for conopeptides, ConoServer, shows that 119 *Conus* species already have at least one protein sequence/structure elucidated. Besides that, this platform shows that conopeptides can be categorized in 12 pharmacological families or in 33 cysteine frameworks. More than 2900 mature conotoxins can be found in this database [136,137].

*Conus* can be classified into three main groups, according to their feeding behavior: worm-hunting, molluscivorous and fish-hunting snails [138]. One of them—*C. regius*—a species that dwells the USA, Central America, and Brazil, including the Fernando de Noronha archipelago, has been studied by these authors [139]. As feeding behavior is often related with venom composition, the authors have investigated what would be the feeding habits of these animal, since they were not known at the time. They found that *C. regius* preferentially preys on fire-worms, thus being categorized as a vermivorous species. Authors have also evaluated the homogeneity of the venom and have determined that, regardless of gender, size and season of the year, there was no significant variation on venom composition (as determined by RP-HPLC peak area and similarity). Under these conditions, they have found the major peak, isolated and characterized it, which led to the identification of rg11a, a conotoxin presenting the cysteine pattern C-C-CC-CC-C-C and ~5 kDa [140]. Later, these authors also described α-RgIB: a 2.7 kDa peptide bearing the CC-C-C pattern, which is an antagonist of neuronal acetylcholine receptor and is capable of inducing hyperactivity in mice and breathing difficulties [141].

### 1.9. Stingrays

Stingrays accounted for 69% of aquatic animal accidents in Brazil from 2007 to 2013. Most cases (88.4%) were reported in the north region and correspond to accidents caused by freshwater stingrays [142].

In general, symptoms of freshwater stingray accidents include skin necrosis, edema, erythema and intense pain, mainly at the lower limbs, which are the most common accident site. Several studies have focused on the mechanism of action of stingray toxins. One explanation is the release of proinflammatory interleukins that lead to the inflammatory reaction and pain, besides the direct participation of mast cell degranulation and histamine release [143,144]. The presence of inflammatory cells in the necrotic tissues was reported, most lymphoid, CD3+ and CD4+ cells, as well as the presence of eosinophils [145].

Although less frequent, marine rays also cause human accidents, but few works report them. In this sense, some of these authors have studied *Hypanus americanum*’s mucus, searching for toxins [146]. It is noteworthy to mention that a marine stingray’s whole body is covered by mucus produced by epithelial cells. Some animals possess a calcified spine (‘sting’) on their tail, which is covered by an epithelium that secretes mucus. This secretion is rich in molecules involved in the chemical defense and skin homeostasis maintenance, including establishing a barrier against microorganisms.

These authors observed that the mucus is labile, denaturating in function of the temperature and storage time after collection. Moreover, the classical scratching method for mucus collection results in the attainment of a mucus rich in cellular debris and, consequently, intracellular content that masks the ‘actual’ mucus. Authors were forced to develop a new method: the whole animal was submerged—for 40 s—in a tank containing only freshwater. After the animal was removed, the water was acidified (0.1% final concentration) and the solution was filtered. This large volume was directly pumped into the C18-RP-HPLC column via system pump ‘A’. After total sample loading, standard chromatography was performed [146].

Nevertheless, the chemical nature of the mucus revealed itself to be more complex than initially imagined by those authors. Several proteins, peptides and low-molecular-mass compounds could be detected. The mucus elicits inflammatory reactions, such as edema and leukocyte recruitment in mice. The performed zymograms displayed proteolytic activity. Moreover, authors describe the antimicrobial effect of molecules fractionated from the mucus. The proteomic analyses revealed proteins that are involved in the immune response, and are very similar to the proteins related to the sting, and also similar to proteins described in fishes from Teleostei class, indicating that the epidermal secretions of stingrays could be more related to an innate immune system than with a venom delivery system [146]. This hypothesis was recently reinforced in a work that analyzed the genomic data of a venomous fish and associated the presence of aerolysin (considered as a toxin) with the immune system [147].

### 1.10. Cnidarians

The phylum Cnidaria comprises more than 10,000 species and is considered the most ancient venomous animal lineage, having emerged approximately 650 million years ago [148,149]. To the contrary of other venomous animals, cnidarians have the unique characteristic of lacking a centralized venom system [150]. Instead of a venom gland, these animals present little organelles distributed throughout their bodies, called cnidaes. Such structures are produced by the Golgi apparatus of specialized cells: the cnidoblasts [151]. It is divided into three main lineages: 1. Anthozoa, formed by Anthozoa class; 2. Medusozoa, comprised of Scyphozoa, Staurozoa, Cubozoa and Hydrozoa classes; 3. Endocnidozoa, comprising Myxozoa and Polypodiozoa classes. Cnidaria is a diverse phylum, rich in bioactive molecules, known to be used mainly for predation, defense and intraspecific competition [152].

Cnidaria early studies began in 1903 on *Anemonia sulcata* and *Actinia equina* tentacles extracts. Since then, several studies on sea anemones have been developed, leading to more than a century of research on these animals’ venoms [150,153,154]. Sea anemones are exquisite sources of toxins and represent the greatest diversity in Anthozoa, having around 1200 species distributed in 46 families [150].

These cnidarians can cause envenomation through their nematocysts, specialized structures that inoculate venom. One particular case report of a human accident caused by anemones belonging to the *Stichodactyla* genus describes local skin irritations with blistering, edema and hemorrhage, mild symptoms when compared to the actual target of the toxin, prey, which is instantly killed by neuro- and cardiotoxins [155]. The anemone toxins molecular scaffolds are diverse: at least 17 different structural motifs are known [150].

The peptide neurotoxins found in sea anemones may act over different ion channels. ShK toxins, for example, bind to Kv type 1; some types of β-defensins can modify the action of Kv type 3 and Nav type 1, 2 and 4; while the inhibitor cystine-knot (ICK) can act over Kv type 5 and acid-sensing ion channels [150]. In this context, a study published in 2004, by some of these authors, investigated the differential selectivity between three sea anemones toxins against a wide range of Nav channels subtypes (Nav 1.1–1.6). The authors observed that for Nav1.3, the three toxins (ATX-II, AFT-II and Bc-III) were active only when at high concentrations. Additionally, it was observed that although ATX-II (from *A. sulcata*) and AFT-II (from *A. fuscoviridis*) exhibit similar sequences, a single amino difference was enough to alter the ion channels specificity. Lysine^36^ (ATX-II) seems to be fundamental for its action over Nav1.1 and Nav1.2 channels; meanwhile, AFT-II mainly exerts effects on Nav 1.4 and Nav 1.5. Moreover, the slight changes in amino acids between similar Nav channels can have a crucial role in toxins binding. For example, AFT-II had a more potent effect over Nav1.4 than Nav 1.5. These two channels are only marginally different and the presence of a Leucine at position 1611 in Nav1.4, instead of an Isoleucine at in Nav1.5 right after a neighboring Asparagine, may indicate the importance of these residues for the toxin binding [156].

In another evaluation of sea anemones venoms, Zaharenko et al. reported, for the first time, the proteomics analysis of the neurotoxic fraction of the sea anemone *Bunodosoma cangicum*. Authors processed by RP-HPLC such a fraction and identified at least 81 different molecules, distributed along 41 chromatographic peaks. Mass spectrometric analysis by MALDI-TOF and ESI-Q-TOF shows that that fraction is composed of low-molecular-mass (280–450 Da) as well as heavier molecules (4–5 kDa). Major fractions were purified and sequenced by Edman degradation, revealing nine novel peptides. Three peptides clearly presented the typical cysteine scaffold found in type 1 sodium channel toxins, and six of them presented new cysteine scaffolds belonging to two new classes of toxins. Additionally, when tested on extracellular crab leg nerve, the new peptides called Bcg31.16 and Bcg30.24 showed that, at very low concentrations (40–50 nM), those neurotoxins were able to diminish the amplitude of CAPs (compound action potentials) and increase its duration, showing a high potency and suggesting that these toxins target sodium channels [157].

Compared to other cnidarians, the Anthozoa (anemones included) is a well-studied group, in terms of toxins investigation. ToxProt lists 256 toxins belonging to 48 species of sea anemones (manually curated; accessed October, 2021). On the other hand, only five toxins from Cubozoa; four from Hydrozoa and one from Scyphozoa classes are deposited [134]. Of particular interest, three Cubozoa toxins (caTX-A, cqTX-A, crTX-A, cfTX-1 and cfTX-2) belong respectively to four species of box jellyfishes: *Carybdea alata*, *Chiropsoides quadrigatus*, *Carybdea rastonii*; and *Chironex fleckeri* (the Australian box jellyfish, one of the most dangerous species of cnidarians) [134,158]. Regardless of the small number of curated toxins, 327 proteins from Cubozoa—computationally analyzed and available at TrEMBL—still remain to be reviewed. The literature refers to Cubozoa toxins being enzymes (phospholipases A2, metallopeptidases and serine peptidases), CRISPs, lectins, pore-forming toxins and protease inhibitors [159]. For Hydrozoa, the four proteins manually curated and described as Hydralysin toxins belong to only two different species: *Hydra viridissima* and *H. vulgaris* [134,135].

The challenge of better knowing the toxins found in Cubozoa and Hydrozoa is not limited to the proteins and peptides; little is known about the low-molecular-mass molecules from these organisms [160]. In order to increase knowledge on the biotechnological potential of Cubozoa and Hydrozoa, two studies were recently performed. The first one, conducted by Bueno et al. [161], investigated the effects of the methanolic extracts of hydromedusa *Olindias sambaquiensis* and jellyfish *Chiropsalmus quadrumanus* over the autonomic neurotransmission. In this study, researchers employed a classical model to sympathetic co-transmission: a myographic evaluation of rat vas deferens bisected in two portions (prostatic and epididymal) for purinergic or adrenergic responses. Throughout the study, both methanolic extracts were demonstrated to be of low complexity and rich in low molecular mass molecules.

Authors report that a low concentration (0.1 μg/mL) of *C. quadrumanus* extract blocked the predominantly noradrenergic contraction of the epididymal end. On the other hand, only high concentrations (1 and 10 μg/mL) of *O. sambaquiensis* extract were capable of leading to the blockade of muscle contraction. Nevertheless, both extracts did not present significant differences concerning the phasic contractions in the prostatic portion (purinergic response), when compared to the control group. Moreover, the histological analysis showed that none of the extracts promote major tissue damage in the prostatic and epididymal vas deferens ends, showing the same unaltered morphology as the control group, which indicates their effects only on the neurotransmission, not causing toxic tissue damages [161].

Another study, published by Arruda et al. [160], focused on *C. quadrumanus* tentacles methanolic extract and its biological activity over neurite growth. In this work, the extract was tested on a human SH-SY5Y neuroblastoma cell line, a neuronal cell culture model commonly used for neurodegenerative disease investigations. Authors report alterations on neurite-related structures of neurons, without affecting cell proliferation or inducing necrosis or apoptosis [160]. The specific neurite length outgrowth observed in all cells exposed to the toxins was associated with a translin-like protein (hyccin cryptein) cryptide, as well as to small molecules acting synergically to promote the neurite/branches formation, elongation and facilitating neurotransmission. Neurite formation can happen either via microtubule and motor proteins [162] or PI4P regulation—acting on plasma membrane identity and myelin development [162]. Moreover, toxins present in the methanolic extract showed no effect on the straightness of neurite’s growth or cell body area, but increased branching junctions connected to cells. More than 14 low molecular mass molecules related to neuritogenesis were found through LC-MS fingerprinting and at least 4 peptides related to neuronal function [160].

### 1.11. Insects

Although insects are the largest group within the arthropod phylum, and most of them are well studied due to their importance, there is always room for new research. Insects are known to create the biological foundation for most of the terrestrial ecosystems by pollinating plants, dispersing seeds, controlling populations of other organisms (including decomposing dead material to recycle nutrients) and being a major food source for other taxa. On the other hand, insects also spread diseases and can compromise a significant amount of food (grains, for example).

Among the notorious insects, there is the honeybee (*Apis mellifera*). There are reports of beekeeping as old as 10,000 years. Bee domestication started in Egypt 4500 years ago, when probably human accidents must have become more frequent, as well.

Bee stinging is mischievous: one single sting may provoke allergy and the subject may die from anaphylaxis. On the other hand, one may be stung several times and, in spite of intense pain and significant swelling, no significant harm occurs. However, when a few dozen bees sting, one may become envenomed. This event is not related to allergy and is a consequence of the bee toxins acting on the victim’s body, especially in the kidneys.

The difference between poison and medicine is the dose, and apitherapy is a rather popular branch of alternative medicine which includes live bee acupuncture. Such a procedure may heal some, but is not free of risks at all! Adverse reactions to bee venom therapy are frequent. Constant exposure to the venom may lead to arthropathy, for example. In sensitized individuals, allergic reactions vary from mild, local swelling to severe systemic reactions, anaphylactic shock and even death. Yet, there are claimed cosmetic uses of the bee venom. Rumor has it that the Duchess of Cambridge has used bee venom to keep her skin looking flawless and even applied the secret ingredient to ensure a glowing complexion when she wed Prince William in 2011.

In a more practical context, a few groups have explored the possibility of developing an antiapilic serum, for treating those patients that have suffered multiple bee stings and have not suffered anaphylactic shock. Among those, authors from this group have successfully developed an efficient antiapilic serum that is currently under clinical trial (phase III). Further details can be found in the works of Ferreira Jr et al., 2010 [163], and Sciani et al., 2010 [164], who set the basis for the preclinical and clinical studies summarized by Barbosa et al., 2021 [165].

## 2. Conclusions

Animal venoms and toxins comprise a diverse repertoire of fascinating proteins, peptides and other bioactive molecules that have evolved through natural selection, driven by adaptive pressure and the survival of the fittest. Their biological role is—mainly—predation and defense. Mankind—and its anthropocentric perspective of nature—have always tried to develop ways to use and study these venoms and toxins as pharmacological prototypes for the research and development of novel therapeutics. Such a quest has opened new venues to the identification of an unprecedented number of new molecules and/or biological effects.

According to our view, ‘classic’ toxinology (as we have termed the continuous study of snakes, scorpions and spiders) will lessen in the near future and the ‘new’ venoms and toxins will prevail, due to subject saturation. Research of unexplored—or neglected—species of animals and their venoms and secretions should become dominant, since they contain a myriad of molecules displaying relevant biological effects on human illnesses, diseases, degenerative disorders, injuries, pain, tumors and infections (viral, bacterial and fungal), either as medicines or diagnostics tools.

Therefore, we consider that the currently reviewed literature on lizards, amphibians, and marine animals is just the beginning of a new thematic approach that we hope will become dominant in the following years. Such veiled potential currently hidden in the neglected animal venoms and toxins can set the instrumental and scientific basis for the development of new molecules with innovative potential, which could shape a “new era” in toxinology.

## Figures and Tables

**Table 1:** Total results retrieved according to the searched terms in different academic databases.

Term	PubMed	Scopus	Web of Knowledge	Google Scholar
Snake	29,272	56,112	44,467	771,000
Scorpion	7030	10,362	8834	91,000
Spider	15,988	42,351	39,343	1,180,000
TOTAL	52,290	108,825	92,644	2,042,000
Amphibian (skin) ^1^	7714	3549	3338	134,000
Sea urchin (toxin) ^2^	314	183	170	19,300
Mollusk ^3^	3688	902	290	19,800
Stingray	813	1717	1817	2160
Cnidarian (toxins)	2389	913	162	17,700
Insects (toxins)	12,879	6663	6037	175,000
TOTAL	27,797	13,927	11,814	367,960
Proportion	1.8×	7.8×	7.8×	5.5×

Search performed in 11 September 2021. ^1^ Limited to skin, in order to exclude ecological studies; ^2^ Limited to toxin, in order to exclude developmental/reproductive models; ^3^ Limited to toxins and excluding dinoflagellates.
